# The Effect of the Preparation Method and the Dispersion and Aspect Ratio of CNTs on the Mechanical and Electrical Properties of Bio-Based Polyamide-4,10/CNT Nanocomposites

**DOI:** 10.3390/polym11122059

**Published:** 2019-12-11

**Authors:** Itziar Otaegi, Nora Aranburu, Maider Iturrondobeitia, Julen Ibarretxe, Gonzalo Guerrica-Echevarría

**Affiliations:** 1 POLYMAT and Polymer Science and Technology Department, Faculty of Chemistry, University of the Basque Country UPV/EHU, Paseo Manuel de Lardizabal 3, 20018 Donostia-San Sebastián, Spain; itziar.otaegi@ehu.eus (I.O.); nora.aramburu@ehu.eus (N.A.); 2 eMERG, School of Engineering of Bilbao, building II-I, University of the Basque Country UPV/EHU, Rafael Moreno Pitxitxi 3, 48013 Bilbao, Spain; maider.iturrondobeitia@ehu.eus (M.I.); julen.ibarretxe@ehu.eus (J.I.)

**Keywords:** aspect ratio, carbon nanotube, dispersion, masterbatch, nanocomposite, polyamide

## Abstract

Bio-based polymeric nanocomposites (NCs) with enhanced electrical conductivity and rigidity were obtained by adding multi-walled carbon nanotubes (CNTs) to a commercial bio-based polyamide 4,10 (PA410). Two different types of commercial CNTs (Cheap Tubes and Nanocyl NC7000^TM^) and two different preparation methods (using CNTs in powder form and a PA6-based masterbatch, respectively) were used to obtain melt-mixed PA410/CNT NCs. The effect of the preparation method as well as the degree of dispersion and aspect ratio of the CNTs on the electrical and mechanical properties of the processed NCs was studied. Superior electrical and mechanical behavior was observed in the Nanocyl CNTs-based NCs due to the enhanced dispersion and higher aspect ratio of the nanotubes. A much more significant reduction in aspect ratio was observed in the Cheap Tubes CNTs than in the Nanocyl CNTs. This was attributed to the fact that the shear stress applied during melt processing reduced the length of the CNTs to similar lengths in all cases, which pointed to the diameter of the CNTs as the key factor determing the properties of the NCs. The PA6 in the ternary PA410/PA6/CNT system led to improved Young’s modulus values because the reinforcing effect of CNTs was greater in PA6 than in PA410.

## 1. Introduction

The study of polymer nanocomposites (NCs), based on carbon nanotubes (CNTs), has attracted significant academic and industrial interest in recent years [1]. Due to their outstanding conductive properties, CNTs make it possible to obtain electrically-conductive thermoplastic/CNT NCs [2], with conductivity values ranging from 10^−8^ to 10^3^ S/cm. Conductivity is achieved when the electrical percolation concentration (p_c_) is reached—i.e., when a three-dimensional network of interconnected nanotubes is formed within the matrix. Furthermore, the addition of CNTs has been widely linked to improvements in the mechanical, thermal, and barrier properties of thermoplastic materials. Therefore, polymer/CNT nanocomposites would seem to be materials of great interest and can potentially be used in leading technological applications such as electrostatic dissipation (ESD) and electromagnetic interference shielding (EMI) [3].

**Figure 1:**
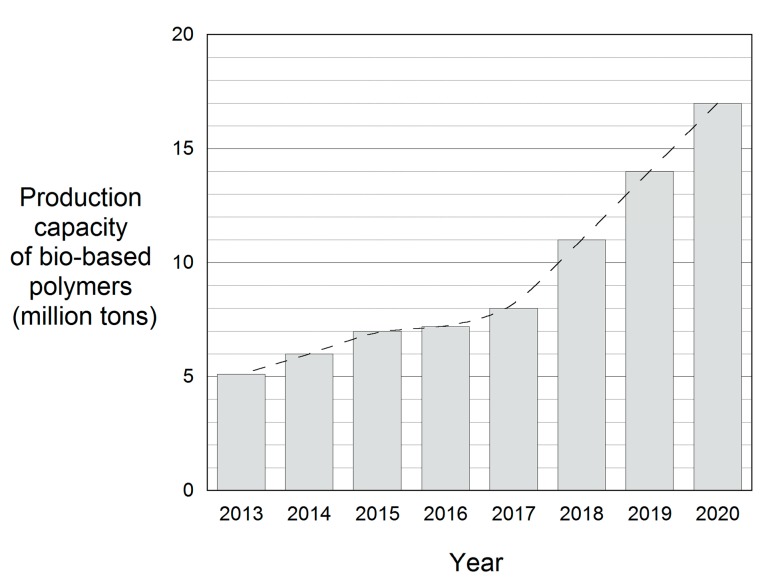
Evolution of the production capacity of bio-based polymers.

Polyamides (PA) have been widely studied in the field of matrices. As they have excellent thermo-mechanical properties, these engineering polymers are widely used in the automotive, packaging, electrical, and electronics industries [4]. Moreover, polyamides from renewable resources (bio-PAs) have recently come onto the market [5,6,7], adding to the wide variety of commercial polyamides [6] already available. Indeed, there has been a growing demand in recent years to replace conventional petrochemical polymers with plastics derived from renewable resources. The production of bio-based polymers is expected to triple from 5.1 million tons in 2013 to 17 million tons in 2020 (Figure 1), and moderate growth is predicted in the bio-polyamides market [6]. The PA410 used in this study is similar to conventional technical polyamides such as PA6 or PA66 in terms of properties, but it is made from bio-based sebacic acid obtained from castor oil [7]. Therefore, its carbon content is approximately 70% renewable, reducing its carbon footprint to almost zero compared with other polyamides. While the butanediamine counterpart used in this study is derived from petroleum, it can also be derived from commercially available bio-based succinic acid or from the direct fermentation of sugars [5].

In the extensive literature on PA/CNT NCs, PA6 is the commercial polyamide most frequently used in the production of this type of nanocomposite [8,9,10]. However, other PAs, such as PA66 [11,12], PA12 [13], PA610 [14], PA1010 [15], and PA11 [16], and to a lesser extent PA46 [17] and PA1212 [18], have also been studied. It is worth mentioning that articles on bio-based PAs are rare [5,19,20] and studies on bio-PA/CNT NCs are practically non-existent.

Regarding the preparation methods of CNT-based nanocomposites, melt mixing is a well-established and cost-effective way of developing new polymeric materials with diverse properties as it is fast, simple, solvent-free, and does not require specific equipment [21]. Therefore, melt mixing is the preparation method of choice in the industry. It is also widely recognized that the orientation, dispersion, and aspect ratio, i.e., the main factors that define the end properties of CNT nanocomposites, are determined by the shear during melt processing [22]. Electrical measurements are commonly used to obtain information about the dispersion of CNTs and the formation of CNT networks, with higher conductivity values indicating well-formed conductive nanotube networks [22]. Accordingly, well-dispersed masterbatches (typically in the range of 10–20 wt% CNT content) are often used as they enable homogeneous dispersions to be obtained. However, a single nanotube parameter is not enough to describe the overall effect on electrical and mechanical properties. Several other parameters need to be considered, such as the length, dispersion, surface quality, and nature of the nanotubes (single, double, or multi-walled carbon nanotubes) [23]. Haggenmueller et al. [24] observed that the single-walled carbon nanotubes composites (SWCNTs) that maintained their length, but exhibited poor dispersion, showed enhanced elastic modulus and electrical conductivity. Finally, the functionalized SWCNTs were better dispersed, but had shorter, more separated nanotubes (caused by the presence of functional groups) and limited mechanical and electrical properties.

The aspect ratios of CNTs play an important role in the percolation threshold for polymer/CNT composites. According to continuum percolation theory, for randomly oriented ideal monodisperse penetrable rods with aspect ratios much greater than one, the following equation can be used to predict the critical percolation (volume) concentration [25,26,27]:

(1) $$\emptyset_{p}=\frac{D}{2L}$$

where $\emptyset_{p}$ is the volume percolation threshold, *D* is the diameter, and *L* is the length of the CNT.

This equation has been found to reasonably describe the experimental data for the electrical conductivity obtained from different multi-walled carbon nanotubes (MWCNTs), as reported by Castillo et al. [26]. In their work, the lowest percolation thresholds recorded for different polycarbonate/MWCNT NCs were attributed to the higher aspect ratios of the corresponding CNTs. Similarly, Guo et al. [25] reported that the electrical percolation threshold of their PC/MWCNT NCs decreased as the CNT aspect ratio increased. Caamaño el al. [27] found that, in their PA66/MWCNT NCs, the percolation thresholds of the larger diameter tubes increased as the L/D ratio decreased. Haggenmueller et al. [24] demonstrated that, at a given CNT concentration, the electrical conductivity of PA66/SWCNT NCs increased with the length of the nanotubes, showing that longer nanotubes are more likely to build percolating paths than shorter nanotubes at a fixed nanotube loading.

Mechanical properties are also affected by the aspect ratio of CNTs. Castillo et al. [26] reported that the storage modulus of PC/MWCNT NCs at 180 and 200 °C increased as the aspect ratio of the MWCNTs increased. Haggenmueller et al. [24] provided evidence that the elastic moduli of nanotube/nylon composites were enhanced at higher nanotube aspect ratios. Other studies have also indicated that the diameter and length, as well as the degree of functionality, of either single-walled or multi-walled CNTs, exert a significant influence on the electrical conductivity, and mechanical and thermal properties of polymer/CNT composites [23,24,27,28].

Therefore, effective mechanical reinforcement and improved electrical properties in CNT-polymer nanocomposites can be achieved with nanotubes with large aspect ratios and good dispersion levels [24]. In the present work, three different commercial bio-PA410/CNT-based systems were prepared and characterized using different CNTs (Cheap Tubes and NC7000^TM^) and different preparation methods (involving CNTs in powder form and a PA6-based CNT masterbatch). Two of the systems shared the same preparation method (CNTs in powder form was melt-blended with the PA410), which allowed for the influence of the different natures of the CNTs to be studied, while the other two shared the same CNTs (NC7000^TM^), which enabled the influence of the preparation method to be examined. Thus, an exhaustive characterization of the aspect ratio of the CNTs employed, comparing the before and after-processing situations, allowed us to evaluate the effect of this parameter on the final properties of the NCs. Furthermore, using a masterbatch is more innovative than using CNTs in powder form because, firstly, masterbatches promote homogeneous CNT dispersion, and secondly, as they are readily available commercially, so the need to use harmful CNT powder is removed from the process, a highly attractive prospect for the polymer industry. This is why the effect of the preparation method on the electrical and mechanical properties of the processed NCs was also studied.

## 2. Materials and Methods

### 2.1. Materials

The PA410 used in this work was EcoPaXX^®^ Q150-D, kindly provided by DSM (Genk, Belgium). Two kinds of commercially available pristine MWCNTs were used: Cheap Tubes (20–30 nm) (Grafton, VT, USA) and Nanocyl NC7000^TM^ (Sambreville, Belgium). Their properties are summarized in Table 1. Additionally, a PA6-based MWCNT masterbatch—also manufactured by Nanocyl (Sambreville, Belgium) and commercialized as Plasticyl^TM^ PA 1503—containing 15 wt % of the aforementioned NC7000^TM^ was also used to obtain the PA410-based NCs.

### 2.2. Composite Processing

All the composites were prepared using the same processing techniques and conditions described in a previous study [29]—it is available as an open access article in this journal. In order to prevent moisture-induced degradation reactions during processing, the PA410 and Plasticyl^TM^ PA 1503 masterbatch were dried in a dry air dehumidifier (Wittmann Drymax, Kottingbrunn, Austria) for 48–72 h at 80 °C. PA410/PA6/CNT NCs were obtained by melt-mixing, using a Collin ZK25 co-rotating twin screw extruder-kneader (Ebersberg, Baviera, Germany) at 270 °C with a screw rotation speed of 200 rpm and diluting the masterbatch with the correct amount of neat PA410. The diameter and length-to-diameter ratios of the screws were 25 mm and 30, respectively. Directly melt-mixed PA410/CNT binary NCs were also obtained under the same conditions, using Cheap Tubes and NC7000^TM^ CNTs. Table 2 shows the wt% content of each component for all the compositions studied.

As described previously [29], the extrudates were cooled in a water bath, pelletized, and dried again. Subsequent injection molding of dried pellets was carried out in a Battenfeld PLUS 350/75 reciprocating screw injection molding machine (Kottingbrunn, Austria) with a press closing force of 350 kN to obtain tensile (ASTM D-638, type IV, thickness 2 mm) specimens. The diameter of the screw in the plasticizing unit was 25 mm and the L/D ratio was 14. The melt and mold temperatures were 270 and 85 °C, respectively. The injection speed, pressure-holding, and cooling times were 42 cm^3^/s, 3 s, and 15 s, respectively. All specimens were kept in a desiccator to prevent post-processing humidity absorption.

Standard circular sheets for electrical conductivity measurements (diameter and thickness: 70 mm and 1 mm, respectively) were obtained by hot pressing in a Collin P200E hydraulic press (Ebersberg, Baviera, Germany). The molding process was carried out at a temperature of 270 °C and a pressure of 130 bar in three stages: Preheating or plasticizing (closure without pressure, 2 min), compression (closure under pressure, 3 min), and cooling under pressure (6 min). Therefore, the only property of the materials in the study that was not measured using injection molded tensile specimens was the conductivity. While injection molding and hot pressing techniques can be expected to produce different CNT dispersion levels, the methodology is still valid when the aim of the measurements is to compare the electrical conductivity of the materials developed using different CNTs and preparation methods.

### 2.3. Phase Structure

The phase behavior was studied by dynamic mechanical analysis (DMA) using a TA Q800 viscoelastometer (New Castle, DE, USA) that provided the loss tangent (tanδ) against temperature. The scans were carried out in single cantilever bending mode at a constant heating rate of 4 °C/min and a frequency of 1 Hz, from −100 to 150 °C. The melting and crystallization behavior of the materials was studied by DSC using a Perkin–Elmer DSC-7 calorimeter (Waltham, MA, USA), which was calibrated using an indium standard as a reference. The samples were first heated from 30 to 300 °C at 20 °C/min and then cooled at the same rate. The melting and crystallization temperatures (T_m_, T_c_) were determined, respectively, from the maxima of the corresponding peaks during the heating and cooling scans, and the melting and crystallization enthalpies were determined from the areas of each of these peaks. The degree of crystallization of PA410 was calculated from the melting and cold crystallization enthalpies, taking the enthalpy of a 100% crystalline ($\Delta H_{f}^{\infty }$) PA410 as 269 J/g [5].

### 2.4. Morphology

The nanostructure was analyzed by transmission electron microscopy (TEM). The samples were obtained from injection-molded specimens and ultrathin-sectioned at ~100 nm using a Leica EMFC 6 ultramicrotome (Wetzlar, Germany) equipped with a diamond knife. The micrographs were obtained in a Tecnai G2 20 twin apparatus (FEI, Waltham, MA, USA), operating at an accelerating voltage of 200 kV.

### 2.5. Characterization of Nanotube Diameter and Length Distributions

The nanotube length and diameter distribution in the composites after processing was analyzed in the three systems under study with 2 wt% CNT content (98/2 in the case of the binary PA410/CNT NCs and 87/11/2 in the case of the ternary PA410/PA6/CNT NCs). The CNTs were extracted as described by Krause et al. [30]. The samples for this characterization were pellets, as received from the extruder after cooling. They were dissolved in formic acid at room temperature for 1 h and the dispersions were treated afterwards for 3 min in an ultrasonic bath. The nanotube concentration in the dispersions was 0.1 g/l.

**Figure 2:**
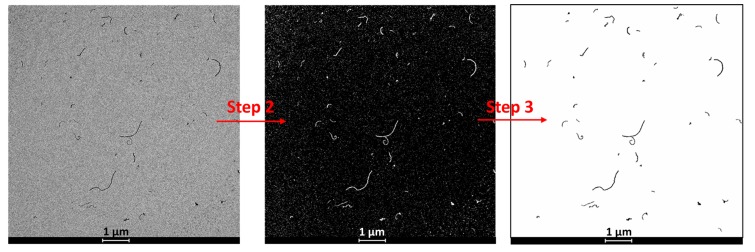
Graphical diagram of Steps 2 and 3 of the image analysis procedure.

For TEM observations, a drop of the newly prepared dispersion was placed onto a glow-discharged, carbon-coated, TEM copper grid (300 mesh) and dried at ambient temperature. TEM micrographs were used for the quantitative characterization of the geometry (length and diameter) of the CNTs. Image analysis was carried out using the free Fiji software [31]. For the characterization of the lengths, TEM micrographs with a pixel size of 2.1 nm/pixel were used. For each sample, 5 TEM micrographs (the number of quantified nanotubes ranged from 223 to 1281, depending on the sample) were analyzed following the procedure described here:

1. The background of the micrographs was homogenized by subtracting the background and applying Gaussian filters.
2. The micrograph was binarized (the objects of interest, the CNTs, were separated from the background). A thresholding method was used for the binarization (Figure 2).
3. Artifacts were removed based on the particle size (Figure 2).
4. Morphological transformations were carried out to improve the quality of the binarized particles.
5. The particles were skeletonized.
6. A quantitative analysis of the skeleton was performed by computing the skeleton length for each particle.

**Figure 3:**
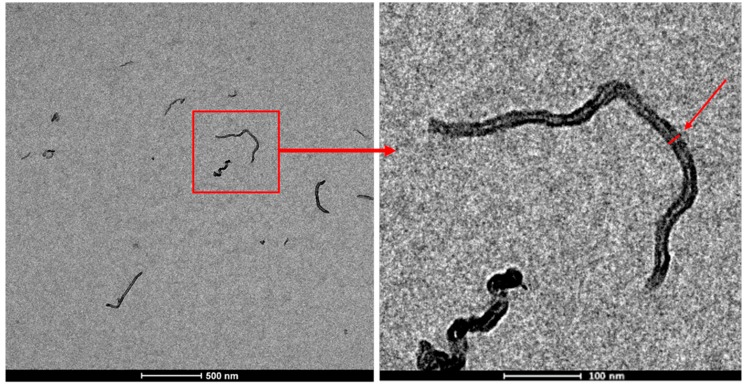
Procedure for nanotube diameter measurements.

The thickness of the nanotubes was measured using higher magnification micrographs with a pixel size of 0.75 nm/pixel (Figure 3). For each sample, 60–70 measurements were performed, using a total of 5 micrographs per sample. Due to their irregular shape, 3–4 measurements were carried out on each nanotube. These measurements were made manually on raw TEM micrographs.

### 2.6. Mechanical Properties

The tensile tests were carried out in an Instron 5569 tensile tester (Instron, Norwood, MA, USA). An extensometer at a crosshead speed of 1 mm/min was used to measure the Young’s modulus. Yield stress and ductility, measured as the break strain (ε_b_), were determined from the load-displacement curves at a crosshead speed of 10 mm/min. A minimum of five tensile specimens were tested for each reported value.

### 2.7. Electrical Properties

The electrical resistivity was determined according to the ASTM D4496-87 standard. Volume resistances were measured and converted to conductivity values. Measurements were taken at 1 V using a Keithley 6487 picoammeter (Cleveland, OH, USA) and a Keithley 8009 Resistivity Test Fixture. Three measurements were performed for each reported value.

## 3. Results and Discussion

### 3.1. Phase Structure

As previously reported [29], the PA410/CNT(1) NCs showed slight T_g_ increases when compared with the neat PA410, due to both the characteristic hindering effect of the CNTs in the movement of polymeric chains in the amorphous phase and the reduction of the free volume. With respect to the crystalline phase, the CNTs did not affect the melting behavior of PA410, and exerted a nucleating effect during cooling, enhancing the overall non-isothermal crystallization rate of the PA410 from the melt. The PA410/CNT(2) NCs, which were not studied in the previous work, showed very similar phase behavior to that of the PA410/CNT(1) NCs (slight increases in T_g_, no effect on the melting behavior and a nucleating effect during cooling).

As described in the previous work [29], since the T_g_ observed for the ternary PA410/PA6/CNT NCs was similar to that of neat PA410 – caused by the CNTs and PA6 offsetting each other, it can be concluded that these ternary NCs are completely miscible in the amorphous phase. With respect to the crystalline phase, a progressive decrease in T_m_ was observed at increasing CNT contents, due to the increase in the PA6 contents. During cooling, the nucleating effect of the CNTs was still evident despite the drop in T_c_ caused by the presence of PA6 in the PA410 matrix. A fully detailed explanation of this result can be found in our previous work [29].

### 3.2. Characterization of Nanotube Diameter and Length Distributions

**Figure 4:**
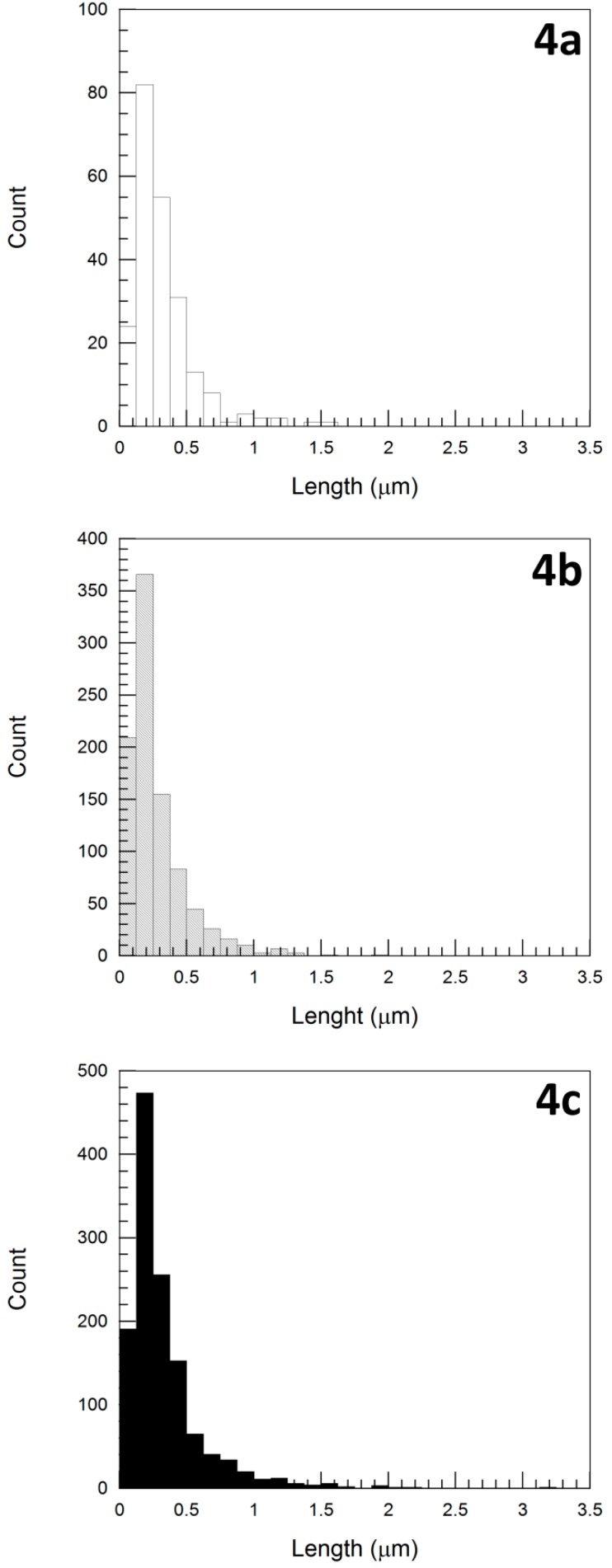
Nanotube length distributions after processing, as recovered from melt extruded PA410/CNT(1) 98/2 (**a**), PA410/CNT(2) 98/2 (**b**), and PA410/PA6/CNT 87/11/2 (**c**) NCs. Total number of particles: 223, 925, and 1281, respectively.

**Figure 5:**
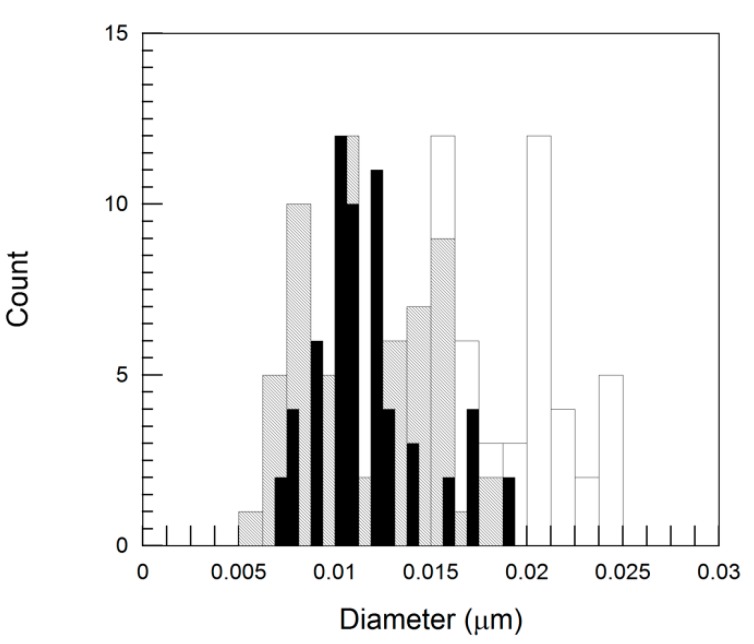
Comparison of diameter distributions of Cheap Tubes (white bars), Nanocyl NC7000^TM^ (grey bars) and Plasticyl^TM^ PA 1503 masterbatch (black bars) after processing, as recovered from melt extruded PA410/CNT(1) 98/2, PA410/CNT(2) 98/2, and PA410/PA6/CNT 87/11/2 NCs. Total number of particles: 65, 60, and 62, respectively (with 3–4 measurements for each nanotube).

Figure 4 and Figure 5 show, respectively, the length and diameter distributions of the CNTs in the three systems, recovered from the melt-extruded NCs after processing, measured from TEM micrographs and following the image analysis procedure described in the experimental part. Table 3 summarizes the experimentally measured average length and diameter values of the different CNTs and the calculated aspect ratio (L/D).

As can be inferred from Figure 4 and seen in Table 3, the average length of the nanotubes in the three systems studied was similar. This result is particularly relevant considering that, before processing, the Cheap Tubes CNTs in the PA410/CNT(1) system were 7–20 times longer (10–30 μm, Table 1) than the Nanocyl CNTs in the PA410/CNT(2) and PA410/PA6/CNT systems (1.5 μm, Table 1). It has previously been demonstrated in the literature that melt processing CNT-based NCs causes the nanotubes to break [22,32] due to the characteristic shear stress in this method of processing. Moreover, as the three systems in the study were obtained under the same processing conditions and therefore subjected to the same shear stress, this result suggests that a certain shear stress reduces the average length of all the CNTs to a similar one, regardless of their initial length. Similar results have previously been obtained in PC-based NCs [25].

With respect to the diameter of the nanotubes, as Figure 5 suggests and Table 3 shows, the values measured by TEM were consistent with those reported in the data sheets (Table 1). Thus, it can be stated that, as expected and unlike its effect on the length, the shear stress applied during melt processing barely affects the diameter of the CNTs.

The aspect ratio of the nanotubes of the three systems, calculated from the length and diameter measurements, is shown in Table 3. As can be seen, the values for the two systems containing Nanocyl CNTs are similar because the length of the CNTs is similar and the diameter is practically identical. However, the aspect ratio of the system containing the Cheap Tubes CNTs is significantly lower than that of the Nanocyl CNT systems, because the length of the Cheap Tubes nanotubes is similar but the diameter is greater. When the experimental L/D values are compared with the values on the data sheets (Table 1), the decrease in the three systems is very significant, but much more so in the PA410/CNT(1) system (95%–99%) than in the systems containing Nanocyl CNTs (82%–84%). Therefore, the high aspect ratio of the Cheap Tubes CNTs before processing was compared with the Nanocyl CNTs drops after processing.

It has been widely reported in the literature and will be demonstrated later in this work that the aspect ratio of carbon nanotubes affects the mechanical and electrical properties of NCs. Many previous studies have also shown that higher aspect ratios result in lower electrical percolation thresholds [25,26], as well as improved mechanical performance.

### 3.3. Morphology

**Figure 6:**
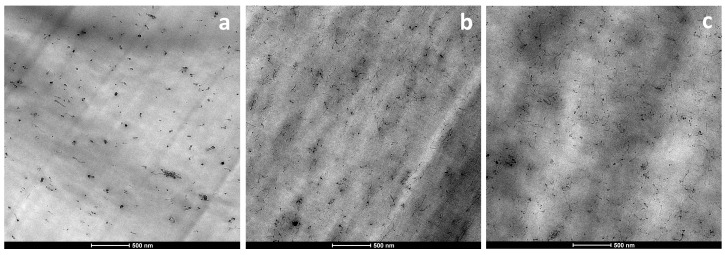
TEM micrographs of the PA410/CNT(1) 99/1 (**a**), PA410/CNT(2) 99/1 (**b**) and PA410/PA6/CNT 93/6/1 (**c**) NCs at x14500 magnification.

Figure 6 shows the TEM micrographs of the PA410/CNT(1), PA410/CNT(2), and PA410/PA6/CNT NCs at a CNT concentration of 1 wt%. As can be seen, the nanotube dispersion levels are qualitatively good in the three NCs under study. Moreover, while difficult to appreciate in the images in Figure 6, a systematic observation of all the TEM micrographs revealed that the nanotube dispersion level of the systems that contained Nanocyl CNTs, i.e., PA410/CNT(2) and PA410/PA6/CNT, was better than the system with the Cheap Tubes CNTs (PA410/CNT(1)). In the former, the nanotubes appeared uncurled and individually dispersed, while in the latter, they were more curled, and there were more small aggregates.

As mentioned in the previous section, the nanotube aspect ratio was significantly higher in the Nanocyl CNTs-based NCs than in the Cheap Tubes CNTs-based ones, due to the similar length but smaller diameter of the nanotubes. Moreover, the density of the pristine Nanocyl CNTs was also lower than that of the Cheap Tubes CNTs. Therefore, given a certain nanofiller wt% content, there were more nanotubes per volume unit in the Nanocyl CNTs-based NCs than in the Cheap Tubes CNTs-based ones.

In conclusion, the dramatic decrease in length—and consequently, in the aspect ratio—of the Cheap Tubes gave rise to lower after-processing aspect ratio values than those of both Nanocyl-based systems. This, along with the greater number and better dispersion of the nanotubes in PA410/CNT(2) and PA410/PA6/CNT NCs, influences both the electrical and mechanical properties of the resulting materials, as will be seen in the following sections.

### 3.4. Electrical Properties

**Figure 7:**
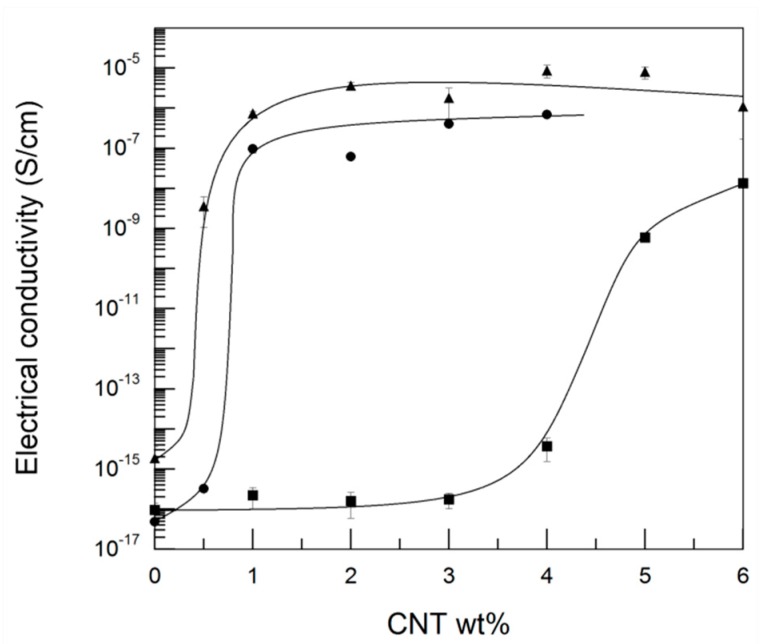
Electrical conductivity of the PA410/CNT(1) NCs (■), the PA410/CNT(2) NCs (▲), and the PA410/PA6/CNT NCs (●). When not visible, the error bars are smaller than the symbols.

Figure 7 shows the electrical conductivity of the PA410/CNT(1), PA410/CNT(2), and PA410/PA6/CNT NCs vs. the CNT content. As mentioned in the experimental section, the specimens for the electrical conductivity measurements were obtained by hot pressing, rather than by injection molding, which was used for the characterization of the other properties in the study. Therefore, as discussed in a previous work [29], different CNT dispersion levels, orientations, and/or even aspect ratios to the injection-molded samples were to be expected. However, given that the samples used for taking the electrical conductivity measurements were all processed in the same way (extrusion followed by compression molding) and under the same conditions, the conductivity results are still valid for the purposes of comparison.

As can be seen in Figure 7, the electrical conductivity increased by over seven decades in the three systems studied, indicating that electrical percolation of the carbon nanofiller occurred. The maximum conductivity values obtained were similar in the PA410/CNT(2) and in the PA410/PA6/CNT systems, but were higher than the figures for the PA410/CNT(1) system. The electrical percolation threshold was calculated from the experimental data using a simple power law Equation (2) [33]:

(2) $$\sigma \left(p\right)=B{\left(p-p_{c}\right)}^{t}$$

where *σ(p)* is the experimental electrical conductivity, *B* is the proportionality constant, *t* is the critical exponent, *p* is the CNT concentration, and *p_c_* is the percolation concentration; where *p* is always higher than *p_c_* (*p* > *p_c_*). The results obtained are stated in Table 4. As can be inferred from Figure 7 and shown in Table 4, the electrical percolation of the carbon nanofiller occurred at similar, significantly lower values in the two Nanocyl CNTs-based NCs than in the Cheap Tubes CNTs-based ones.

The three main factors that affect the electrical behavior of a CNT-reinforced polymeric system are: (1) Orientation, (2) geometry, usually characterized by the aspect ratio, and (3) dispersion level, which encompasses the number of individual CNTs, the aggregates, and the curled or uncurled geometry. The first factor can be excluded because TEM observations clearly show randomly oriented CNTs in the three systems of the study. With respect to the other two, it was previously demonstrated that the aspect ratio and the dispersion level were similar in the PA410/CNT(2) and PA410/PA6/CNT NCs, but higher and better, respectively, than in the PA410/CNT(1) NCs. Thus, the superior electrical performance of the Nanocyl CNT-based NCs compared with the Cheap Tubes CNT-based ones can be attributed to their good dispersion level and higher aspect ratio.

When these experimental percolation concentrations are compared with the theoretical values obtained using Equation (1), calculated using the L and D values provided in the data sheets, it can be seen that the experimental percolation thresholds (Table 4) are much higher than the theoretical values (0.06–0.29 wt%) in the PA410/CNT(1) NCs, and similar or slightly higher than the latter results (0.51 wt%) in the PA410/CNT(2) and PA410/PA6/CNT NCs. The difference in values can thus be attributed to the reduction in the aspect ratio of the different CNTs during the processing referred to earlier. Consistent with the aforementioned data regarding the reduction in the aspect ratio, the maximum difference between the experimental and theoretical percolation values was observed in the PA410/CNT(1) NCs with the Cheap Tubes CNTs.

However, when the theoretical *p_c_* values from Equation (1) were calculated using the experimentally measured L and D values, i.e., after processing, the results were higher (5,17, 3,31, and 3,29 wt%, respectively, for PA410/CNT(1), PA410/CNT(2), and PA410/PA6/CNT NCs) than the experimental p_c_-s shown in Table 4. Given that Equation (1) assumes randomly oriented ideal monodisperse penetrable rods, the lower experimental values obtained in this work are unexpected and point to the experimental methodology used as underestimating the real L/D ratios of the CNTs. It is notable that Equation (1) is consistent with experimentally measured *p_c_* values in certain polymer/CNT systems [25,26], however, as calculations for these systems were performed with the initial L/D values of the CNTs, the effect of the melt-mixing processing on the aspect ratio was not considered in any of these studies.

Finally, it is notable that careful selection of the CNTs brought about some of the lowest *p_c_* values reported in the literature for melt-processed PA-based NCs, with obtained values ranging from 0.4% [34] to 6% [35], depending on the PA, processing method, and conditions used. As previously demonstrated, given that the shear stress applied during processing reduced the initial dissimilar lengths of the CNTs to similar lengths, the initial diameter of the CNTs, which was unchanged by processing, plays a key role in the final aspect ratio of the CNTs, and therefore in the electrical behavior of the NCs.

### 3.5. Mechanical Properties

**Figure 8:**
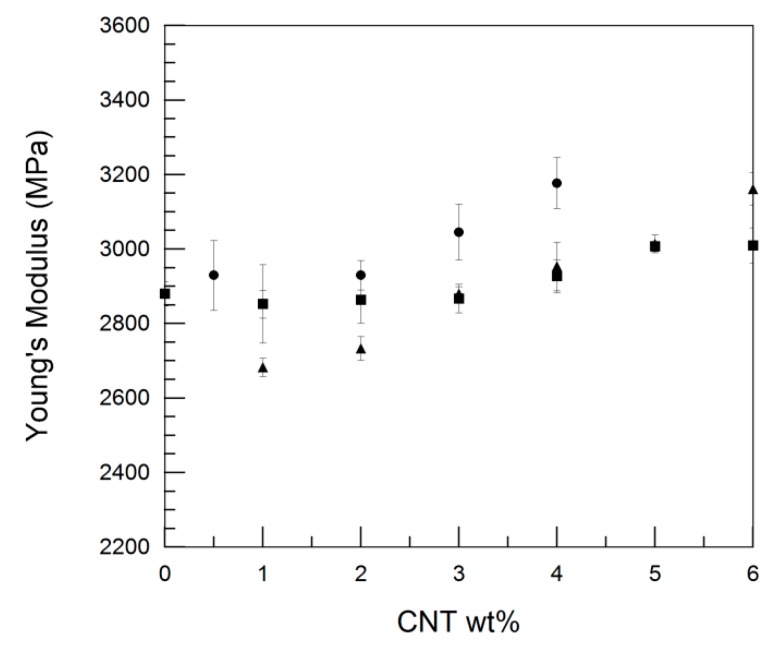
Young’s modulus of the PA410/CNT(1) NCs (■), PA410/CNT(2) NCs (▲), and PA410/PA6/CNT NCs(●).

Table 5 shows the Young’s modulus, tensile strength, and ductility values of the PA410/CNT(1), PA410/CNT(2), and PA410/PA6/CNT NCs. Young’s modulus vs. the CNT content is also shown in Figure 8. As expected in well-dispersed CNT-filled NCs, the stiffness increased at increasing CNT contents in all the NC systems. The overall increase in Young’s modulus is caused by a decrease in the molecular mobility of the polymeric chains, which is also supported by the T_g_ increase shown by DMTA. The large interfacial area-to-dispersed phase volume ratio characteristic of well-dispersed CNTs facilitates the presence of interactions between the polymer and the filler [36]. When the three systems were compared, the maximum modulus increase was observed in the PA410/PA6/CNT system, where a 10.3% increase was observed with a 4 wt% CNT content, followed by the PA410/CNT(2) system (an increase of 11% with a 6 wt% CNT content), and the PA410/CNT(1) system, where a 4.5% increase with a 6 wt% CNT content was observed. Therefore, the reinforcing efficiency of the Nanocyl CNTs is clearly greater than that of the Cheap Tubes CNTs, and can be attributed to the higher aspect ratio and better dispersion rate of the former, as previously discussed.

When comparing the two Nanocyl CNTs-based NCs, the superior mechanical performance of the PA410/PA6/CNT NCs is particularly noteworthy, considering that the Young’s modulus of PA6 (≈2400 MPa) is significantly lower than that of neat PA410 (≈2900 MPa). As mentioned before regarding conductivity, the most important factors affecting the mechanical behavior of CNT-filled NCs are orientation, aspect ratio, and dispersion level. However, these parameters were found to be similar in both the Nanocyl CNTs-based NCs in this study. In fact, the only difference between the two systems is the presence of a certain amount of PA6 in the PA410/PA6/CNT NCs. In the literature, the reinforcing capacity of the Nanocyl CNTs was better in a PA6 matrix [36] than in the PA410 matrix of the present work. This is because the PA6/CNT masterbatch used here was used in a pure PA6 matrix, and a 36% increase in modulus was reported after 5% CNTs were added. In the present work, the addition of the same CNT content to PA410 led to a modulus increase of only 4.5%. As already explained in a previous work [29], the PA410/PA6/CNT NCs studied in the present work were prepared by diluting the aforementioned PA6/CNT masterbatch with the appropriate amounts of pure PA410, while the PA410/CNT(2) NCs were prepared by direct melt-mixing pristine Nanocyl CNTs and PA410. Consequently, the presence of a certain amount of PA6 in the ternary system produces a synergistic reinforcing effect of the CNTs, which, in turn, produces higher Young’s modulus values in PA410/PA6/CNT NCs.

As can be seen in Table 5, the behavior of the yield stress was similar to that of Young’s modulus in the binary PA410/CNT(1) and PA410/CNT(2) NCs, showing slight and moderate increases as the CNT content increased. Similar behavior has been observed in other CNT-filled NCs [9,13,37], although the local nature of the yielding process—unlike the Young’s modulus where the whole section of the specimen is involved—often leads to constant [8,38] or even decreasing [8,39] values at increasing CNT contents. This seems to be the case of the ternary PA410/PA6/CNT system, where the presence of PA6, with its lower yield stress (≈66 MPa), coupled with the local nature of the yielding process lead to values similar to that of neat PA410.

The ductility of all the NC systems in the study decreased sharply even at the lowest CNT contents, as expected in CNT-reinforced NCs. This decrease is attributed to restrictions in the mobility of the matrix chains caused by the single CNTs, which promote fracture, and to the intrinsic crack-sensitive nature of PAs [40,41,42]. No relevant differences were observed among them, and all the filled compositions broke just after yielding.

## 4. Conclusions

Bio-based polymeric NCs with enhanced electrical conductivity and rigidity were obtained by adding MWCNTs to PA410. Superior electrical and mechanical behavior was observed in the Nanocyl CNTs-based NCs due to the improved dispersion and higher aspect ratio of the nanotubes. A much more significant reduction in aspect ratio was observed in the Cheap Tubes CNTs compared to the Nanocyl CNTs. This is attributed to the fact that the shear applied during melt processing reduced the length of the CNTs to a similar value in all cases, pointing to the diameter of the CNTs as the key factor determining the properties of the NCs. The presence of PA6 in the ternary PA410/PA6/CNT system led to improved Young’s modulus values because the CNTs had greater reinforcing ability in PA6 than in PA410.

## Figures and Tables

**Table 1:** Properties of the multi-walled carbon nanotubes (MWCNTs) used, according to their respective data sheets.

Sample	Diameter (nm)	Length (μm)	Estimated Aspect Ratio (L/D)	Surface Area (m^2^/g)	Carbon Purity (%)
Cheap Tubes ^1^	20–30	10–30	333–1500	110	>95
Nanocyl ^2^	9.5	1.5	158	250–300	90

^1^ Cheap Tubes. Description Multi Walled Carbon Nanotubes 20–30 nm; ^2^ Nanocyl. Technical Data Sheet: NC7000^TM^, 12th July 2016, V08.

**Table 2:** PA410, PA6, and carbon nanotubes (CNT) wt % content in the compositions studied.

	Nomenclature	PA410 (wt %)	PA6 (wt %)	Cheap Tubes (wt %)	Nanocyl NC7000^TM^ (wt %)
PA410/CNT(1) binary NCs	100/0	100	-	0	-
	99/1	99	-	1	-
	98/2	98	-	2	-
	97/3	97	-	3	-
	96/4	96	-	4	-
	95/5	95	-	5	-
	94/6	94	-	6	-
PA410/CNT(2) binary NCs	99.5/0.5	99.5	-	-	0.5
	99/1	99	-	-	1
	98/2	98	-	-	2
	97/3	97	-	-	3
	96/4	96	-	-	4
	95/5	95	-	-	5
	94/6	94	-	-	6
PA410/PA6/CNT ternary NCs	96.6/2.9/0.5	96.6	2.9	-	0.5
	93/6/1	93	6	-	1
	87/11/2	87	11	-	2
	80/17/3	80	17	-	3
	73/23/4	73	23	-	4

**Table 3:** Average values of the diameter and length, measured from transmission electron microscopy (TEM) micrographs, and estimated aspect ratio of the CNTs after processing the NCs.

NC	Diameter (nm)	Length (μm)	Aspect ratio (L/D)
PA410/CNT(1)	18	0.32	18
PA410/CNT(2)	11	0.27	25
PA410/PA6/CNT	12	0.33	28

**Table 4:** Experimental percolation thresholds of the NCs.

NC.	Percolation Threshold (wt%)
PA410/CNT(1)	3.98
PA410/CNT(2)	0.50
PA410/PA6/CNT	0.65

**Table 5:** Young’s modulus, tensile strength and ductility values of the PA410/CNT(1), PA410/CNT(2), and PA410/PA6/CNT NCs.

	Composition	Young’s Modulus (MPa)	Tensile Strength (MPa)	Strain at Break (%)
PA410/CNT(1) NCs	100/0	2880 ± 30	75.5 ± 0.4	100 ± 30
	99/1	2850 ± 100	74.3 ± 1.2	7 ± 4
	98/2	2860 ± 60	75.6 ± 0.2	7 ± 1
	97/3	2870 ± 40	74.3 ± 1.2	5 ± 1
	96/4	2930 ± 40	75.1 ± 0.7	7 ± 1
	95/5	3010 ± 10	74.5 ± 0.8	5 ± 1
	94/6	3010 ± 50	76.3 ± 0.5	6 ± 0
PA410/CNT(2) NCs	99.5/0.5	2630 ± 10	75.8 ± 0.3	14 ± 6
	99/1	2680 ± 20	76.5 ± 3.4	5 ± 2
	98/2	2730± 30	79.2 ± 0.4	6 ± 4
	97/3	2880 ± 20	83.2 ± 0.4	9 ± 5
	96/4	2950 ± 60	82.6 ± 1.8	7 ± 4
	95/5	3010 ± 20	85.8 ± 0.9	8 ± 1
	94/6	3160 ± 40	84.5 ± 1.0	4 ± 1
PA410/PA6/CNT NCs	96.6/2.9/0.5	2930 ± 90	74.3 ± 0.3	8 ± 4
	93/6/1	2850 ± 40	73.5 ± 0.5	5 ± 1
	87/11/2	2930 ± 40	73.8 ± 1.0	6 ± 2
	80/17/3	3040 ± 80	71.8 ± 3.6	4 ± 1
	73/23/4	3180 ± 70	74.1 ± 2.0	3 ± 0
